# Icariin attenuates angiotensin II-induced hypertrophy and apoptosis in H9c2 cardiomyocytes by inhibiting reactive oxygen species-dependent JNK and p38 pathways

**DOI:** 10.3892/etm.2014.1598

**Published:** 2014-03-04

**Authors:** HENG ZHOU, YUAN YUAN, YUAN LIU, WEI DENG, JING ZONG, ZHOU-YAN BIAN, JIA DAI, QI-ZHU TANG

**Affiliations:** 1 Department of Cardiology, Renmin Hospital, Wuhan University, Wuhan, Hubei 430060, P.R. China; 2 Cardiovascular Research Institute, Wuhan University, Wuhan, Hubei 430060, P.R. China

**Keywords:** icariin, hypertrophy, apoptosis, reactive oxygen species, mitogen activated protein kinases

## Abstract

Icariin, the major active component isolated from plants of the Epimedium family, has been reported to have potential protective effects on the cardiovascular system. However, it is not known whether icariin has a direct effect on angiotensin II (Ang II)-induced cardiomyocyte enlargement and apoptosis. In the present study, embryonic rat heart-derived H9c2 cells were stimulated by Ang II, with or without icariin administration. Icariin treatment was found to attenuate the Ang II-induced increase in mRNA expression levels of hypertrophic markers, including atrial natriuretic peptide and B-type natriuretic peptide, in a concentration-dependent manner. The cell surface area of Ang II-treated H9c2 cells also decreased with icariin administration. Furthermore, icariin repressed Ang II-induced cell apoptosis and protein expression levels of Bax and cleaved-caspase 3, while the expression of Bcl-2 was increased by icariin. In addition, 2′,7′-dichlorofluorescein diacetate incubation revealed that icariin inhibited the production of intracellular reactive oxygen species (ROS), which were stimulated by Ang II. Phosphorylation of c-Jun N-terminal kinase (JNK) and p38 in Ang II-treated H9c2 cells was blocked by icariin. Therefore, the results of the present study indicated that icariin protected H9c2 cardiomyocytes from Ang II-induced hypertrophy and apoptosis by inhibiting the ROS-dependent JNK and p38 pathways.

## Introduction

Cardiac hypertrophy occurs when the heart endures overload or injury. Although hypertrophy is an adaptive process that initially maintains cardiac output, sustained hypertrophy ultimately leads to heart failure, which is the leading cause of morbidity and mortality worldwide (1,2). Various stimuli, including mechanical stress and neurohumoral factors, such as angiotensin II (Ang II), endothelin-1, catecholamine and growth factors, are involved in the progression of cardiac hypertrophy (3). These stimuli activate membrane receptors and intracellular signaling pathways to mediate the transcription of hypertrophy-related genes (4). Enlargement and apoptotic loss of cardiomyocytes are the key pathological changes in cardiac hypertrophy (5). To prevent cardiomyocytes from enlargement and cell death, blocking the transition between adaptive hypertrophy and heart failure is necessary. However, the existing treatments that modify hemodynamics and inhibit active neurohumoral factors are not capable of successfully restoring the injured cardiomyocytes. Disrupting the intracellular signaling pathways that mediate cardiac hypertrophy has been increasingly studied and novel targets have been identified that may be used to explore therapeutic strategies for cardiac hypertrophy and heart failure.

Icariin (C_33_H_40_O_15_; molecular weight, 676.66), a prenylated flavonol glycoside, is the major active component isolated from plants of the Epimedium family (6). Multiple pharmacological properties of icariin have been revealed, including immunoregulation, antioxidative stress, antiapoptosis and stimulation of angiogenesis (6–9). Song *et al* (8) identified that icariin attenuated cardiac remodeling in rats with congestive heart failure by inhibiting matrix metalloproteinase (MMP) activity and protecting cardiomyocytes from apoptosis. This result demonstrates the cardiac protective role of icariin. However, it is not known whether icariin has a direct effect on cardiomyocytes and the mechanism underlying its cardiac protective role remains unclear.

Ang II functions as a significant hormonal mediator in cardiac hypertrophy that can induce a direct injury on cardiomyocytes. Reactive oxygen species (ROS)-dependent activation of the c-Jun N-terminal kinase (JNK) and p38 pathways has been shown to play a critical role in the effect Ang II exhibits on cardiomyocytes (10). A previous study demonstrated that icariin inhibits the production of ROS and blocks the activity of the JNK and p38 pathways in lipopolysaccaride (LPS)-treated microglial cells (11). However, the effect of icariin on Ang II-induced cardiomyocyte injury and the underlying mechanisms remain unknown. In the present study, a hypertrophic model was used in Ang II-stimulated H9c2 cardiomyocytes. The aims were to determine whether icariin treatment directly prevented cardiomyocytes from hypertrophy and apoptosis and to determine whether the cardioprotective effect of icariin was mediated via the inhibition of the ROS-dependent JNK and p38 pathways.

## Materials and methods

### Reagents

Icariin (≥94% purity as determined by high performance liquid chromatography analysis), Ang II and 2′,7′-dichlorofluorescein diacetate (DCFH-DA) were purchased from Sigma-Aldrich (St. Louis, MO, USA). Dulbecco’s modified Eagle’s medium: Nutrient mixture F-12 (DMEM/F12), fetal bovine serum (FBS), trypsin, penicillin and streptomycin were purchased from Gibco-BRL (Carlsbad, CA, USA). TRIzol, Alexa Fluor^®^ 488 goat anti-mouse IgG and SlowFade Gold antifade reagent with 4′,6-diamidino-2-phenylindole (DAPI) were purchased from Invitrogen Life Technologies (Carlsbad, CA, USA). A Transcriptor First Strand cDNA synthesis kit and Light Cycler 480 SYBR Green 1 Master Mix were purchased from Roche Diagnostics (Basel, Switzerland). Antibodies against α-actinin and an ApopTag^®^ Plus Fluorescein In Situ Apoptosis detection kit were purchased from Millipore Corporation (Billerica, MA, USA). Primary antibodies were purchased from Cell Signaling Technology, Inc. (Beverley, MA, USA) and IRDye 800CW conjugated secondary antibodies were obtained from LI-COR Biosciences (Lincoln, NE, USA).

### H9c2 cardiomyocyte culture

The H9c2 embryonic rat heart-derived cell line was obtained from the Cell Bank of the Chinese Academy of Sciences (Shanghai, China). Icariin was dissolved in dimethyl sulfoxide at a concentration of 10 mmol/l for storage. Cells were cultured in DMEM/F12 1:1 medium, supplemented with 10% FBS, 100 U/ml penicillin and 100 mg/ml streptomycin, in a humidified incubator with an atmosphere of 5% CO_2_ at 37°C. Cells were seeded at a density of 1×10^6^ cells per well into six-well culture plates for mRNA extraction, 5×10^5^ cells per well into six-well culture plates for cell surface area (CSA) and terminal deoxynucleotidyl transferase-mediated dUTP nick end-labeling (TUNEL) analysis, 5×10^3^ cells per well in 96-well plates for ROS detection and 1×10^7^ cells per well into 100 mm culture dishes for protein extraction. The cells were cultured in serum-free DMEM/F12 1:1 medium for 24 h and pretreated with icariin for 1 h prior to stimulation with Ang II.

### Cell viability

Cell viability was analyzed using the Cell Counting Kit-8 (CCK-8) assay. Following icariin treatment for 48 h, 10 μl CCK-8 solution was added to each well of the 96-well plate and then incubated for an additional 4 h. Absorbance was measured at 450 nm using a microplate reader (Synergy HT; BioTek, Winooski, VT, USA). The percentage of cell viability was calculated according to the following formula: Cell viability (%) = optical density (OD) of the treatment group/OD of the control group × 100%.

### Quantitative polymerase chain reaction (qPCR)

To detect the mRNA expression levels of hypertrophic markers, including atrial natriuretic peptide (ANP) and B-type natriuretic peptide (BNP), qPCR was performed as described previously (12). Total RNA was extracted from cultured H9c2 cells using TRIzol and 2-μg samples of RNA were reverse-transcribed into cDNA using the Transcriptor First Strand cDNA synthesis kit. PCR amplifications were quantified using a LightCycler 480 SYBR Green 1 Master Mix and GAPDH was used as the internal control.

### CSA analysis

To assess CSA, cells were stained by immunofluorescence for cardiac α-actinin (13). The cells were washed with phosphate-buffered saline (PBS), fixed with RCL2 fixing liquid and permeabilized in 0.1% Triton X-100 in PBS. The cells were stained with anti-α-actinin at a dilution of 1:100 in 1% goat serum overnight at 4°C, and then incubated with Alexa Fluor^®^ 488 goat anti-mouse IgG for 1 h at 37°C. Cells on the coverslips were mounted onto glass slides with SlowFade Gold antifade reagent with DAPI and CSAs were measured using a quantitative digital image analysis system (Image Pro-Plus version 6.0; Media Cybernetics, Inc., Rockville, MD, USA).

### TUNEL staining

Apoptotic nuclei were labeled using TUNEL staining with a ApopTag^®^ Plus Fluorescein In Situ Apoptosis Detection kit, according to the manufacturer’s instructions (14). Cells on the coverslips were fixed with 1% paraformaldehyde in PBS, stained with TUNEL reagents and the nuclei were stained with DAPI. The cell apoptotic index was calculated as the percentage of apoptotic nuclei/total number of nuclei.

### ROS detection

Intracellular ROS generation was determined using DCFH-DA, which becomes fluorescent on oxidation to DCF by H_2_O_2_ produced within cells. Following Ang II or/and Icariin treatments, H9c2 cells were washed twice and incubated with 5 μM DCFH-DA solution in serum-free medium at 37°C for 30 min in the dark. Data were then collected using a fluorescent reader (Synergy HT; BioTek) at excitation/emission wavelengths of 485/530 nm. A fluorescent microscope was also used to evaluate the DCF fluorescence of the cells on the coverslips.

### Western blotting

Western blotting was performed as described previously (13). Cells were lysed in radioimmunoprecipitation assay lysis buffer and 50-μg samples of the cell lysates were electrophoresed on 10% SDS-PAGE gels. The proteins were then transferred onto Immobilon-FL transfer membranes (Millipore Corporation) and blocked with 5% non-fat milk for 2 h. The membranes were incubated with antibodies specific for phosphorylated (p)-JNK, p-extracellular signal-related kinase (ERK), p-p38, total (T)-JNK, T-ERK, T-p38, Bcl-2, Bax, cleaved-caspase 3 or GAPDH overnight at 4°C. The samples were then incubated with IRDye 800CW conjugated secondary antibodies and the blots were scanned by a two-color infrared imaging system (Odyssey, LI-COR Biosciences).

### Statistical analysis

Data are presented as the mean ± SEM and analyzed using a statistical software (SPSS 16.0; SPSS Inc., Chicago, IL, USA). Differences among the groups were determined by two-way analysis of variance followed by Tukey’s post hoc test. A comparison between the control and all the treatment groups was performed using the unpaired Student’s t-test. P<0.05 was considered to indicate a statistically significant difference.

## Results

### Effect of icariin on cell viability

**Figure 1:**
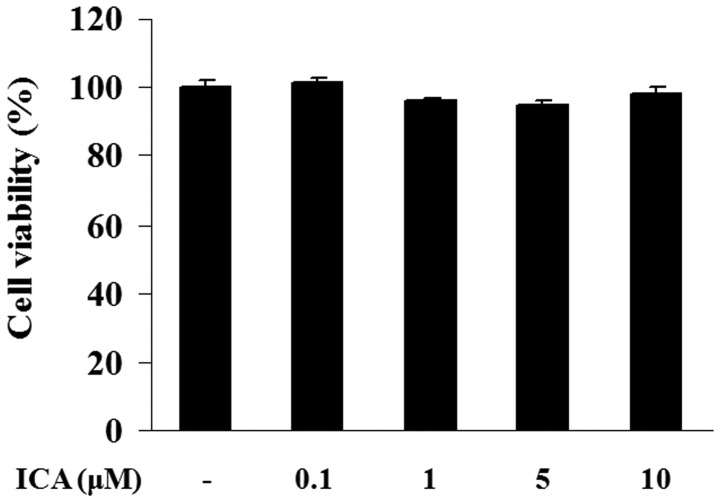
Effect of icariin on cell viability, as determined using the CCK-8 assay. Treatment with the indicated concentrations of icariin for 48 h did not cause any significant change in cell viability when compared with the control group. CCK-8, cell counting kit-8.

The potential cytotoxicity of icariin was analyzed using a CCK-8 assay. H9c2 cells were incubated with various concentrations of icariin (0.1, 1, 5 or 10 μM) for 48 h. Cell viability in icariin-treated cells exhibited no significant differences when compared with the control cells, indicating that icariin at a concentration of 0.1, 1, 5 or 10 μM did not possess any cytotoxicity in H9c2 cells (Fig. 1).

### Effect of icariin on ANP and BNP induction

**Figure 2:**
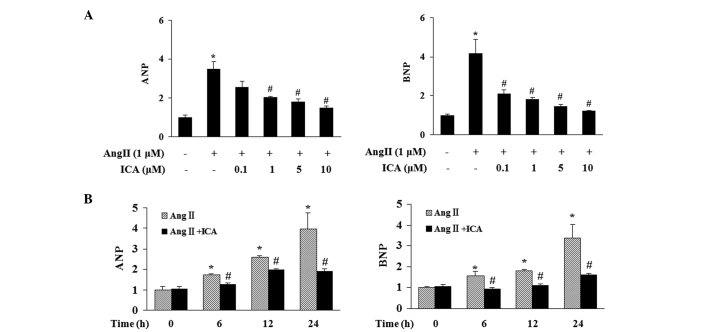
Effect of icariin on the mRNA expression levels of ANP and BNP. (A) Icariin decreased Ang II-induced mRNA expression levels of ANP and BNP in a concentration-dependent manner. ^*^P<0.05, vs. control; ^#^P<0.05, vs. Ang II-treated cells. (B) Icariin (10 μM) decreased Ang II (1 μM)-induced mRNA expression levels of ANP and BNP at the indicated time points. ^*^P<0.05, vs. cells at 0 time; ^#^P<0.05, vs. Ang II-treated cells at 0 time. Ang II, angiotensin II; ANP, atrial natriuretic peptide; BNP, B-type natriuretic peptide.

The effect of icariin at various concentrations (0.1, 1, 5 or 10 μM) on the induction of ANP and BNP in response to Ang II was determined. Stimulation with Ang II for 24 h markedly increased the mRNA expression levels of ANP and BNP in H9c2 cells and icariin treatment markedly attenuated this increase in a concentration-dependent manner (Fig. 2A). Icariin at a concentration of 10 μM significantly blocked the induction of ANP and BNP in response to Ang II at various time points, thus, this concentration was selected for further investigations (Fig. 2B).

### Icariin attenuates the Ang II-induced increase in CSA

**Figure 3:**
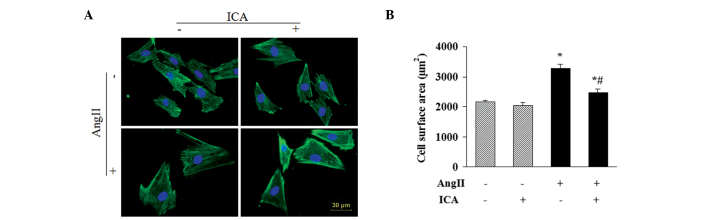
Effect of icariin on CSA. (A) Representative images and (B) quantitative results demonstrating that 10 μM icariin inhibited Ang II (1 μM; 48 h)-induced enlargement of H9c2 cells. ^*^P<0.05, vs. control; ^#^P<0.05, vs. Ang II-treated cells. CSA, cell surface area; Ang II, angiotensin II.

CSAs of H9c2 cells were determined by α-actinin staining to further evaluate the antihypertrophic effect of icariin. Ang II stimulation for 48 h resulted in a significant increase in the CSAs of H9c2 cells. However, icariin treatment markedly attenuated the increase, indicating that Ang II-induced enlargement of H9c2 cells was suppressed by icariin (Fig. 3).

### Icariin inhibits Ang II-induced apoptosis

**Figure 4:**
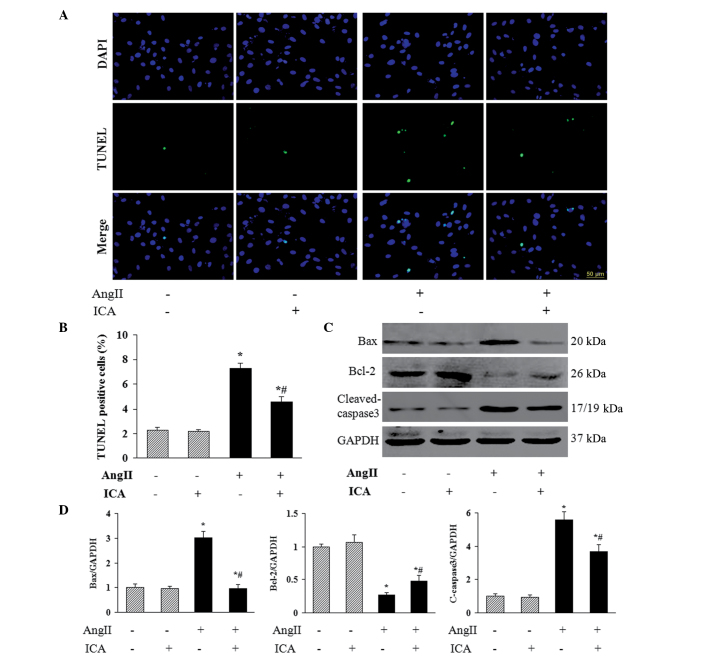
Effect of icariin on apoptosis. (A) Representative images and (B) quantitative results demonstrating that 10 μM icariin attenuated cell apoptosis following stimulation with Ang II for 48 h, as shown by TUNEL staining. (C) Representative blots and (D) quantitative results demonstrating that 10 μM icariin decreased the protein expression levels of Bax and cleaved-caspase 3, but increases the expression levels of Bcl-2 in response to Ang II stimulation. ^*^P<0.05, vs. control; ^#^P<0.05, vs. Ang II-treated cells. Ang II, angiotensin II; TUNEL, terminal deoxynucleotidyl transferase-mediated dUTP nick end-labeling.

To investigate the role of icariin in Ang II-induced apoptosis of H9c2 cells, TUNEL staining was used to identify the apoptotic nuclei. A marked increase in the number of TUNEL-positive nuclei was observed in cells that had been incubated with Ang II, and icariin treatment markedly reduced Ang II-induced cell apoptosis (Fig. 4A and B). In addition, icariin decreased the protein expression levels of Bax and cleaved-caspase 3 in H9c2 cells in response to Ang II (Fig. 4C and D), which may mediate the antiapoptotic effect of icariin. Furthermore, the decreased level of antiapoptotic protein Bcl-2 in Ang II-treated H9c2 cells was restored with icariin treatment (Fig. 4C and D).

### Icariin decreases the production of ROS

**Figure 5:**
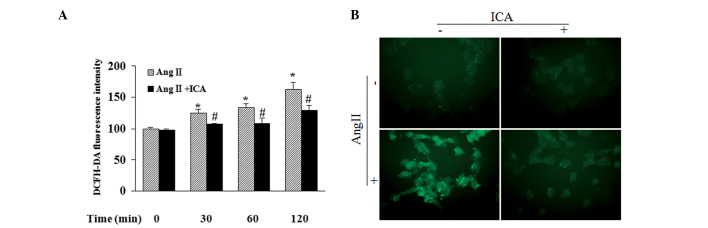
Effect of icariin on ROS production. (A) Icariin (10 μM) blocked 1 μM Ang II-induced ROS production at the indicated time points, as detected by a fluorescent reader. (B) Fluorescent microscope images demonstrating that 10 μM icariin attenuated DCF-derived fluorescence in cells treated with 1 μM Ang II for 2 h. ^*^P<0.05, vs. cells at 0 time; ^#^P<0.05, vs. Ang II-treated cells at 0 time. ROS, reactive oxygen species; Ang II, angiotensin II; DCF, 2′7′-dichlorofluorescein.

Fluorescence intensity in cells following incubation with DCFH-DA, exhibited by a fluorescent reader, revealed that Ang II increased the ROS content in a time-dependent manner. In addition, icariin treatment markedly blocked Ang II-induced ROS production at the indicated time points (Fig. 5A). DCF-derived fluorescence observed with a microscope also demonstrated that icariin inhibited the accumulation of intracellular ROS in Ang II-treated cells, which was in accordance with the results of the fluorescent reader (Fig. 5B).

### Icariin blocks the activation of JNK and p38 pathways in response to Ang II

**Figure 6:**
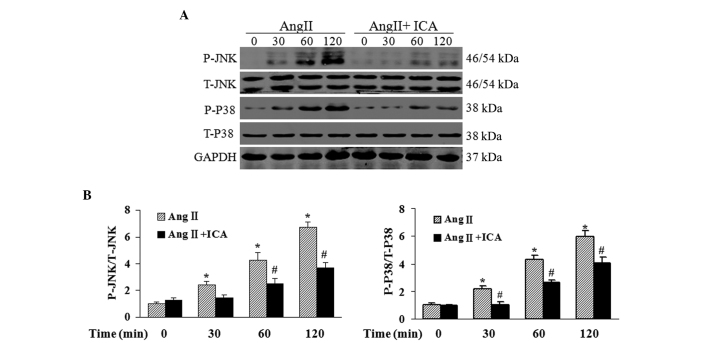
Effect of icariin on the activation of JNK and p38 pathways. (A) Representative blots and (B) quantitative results demonstrating that icariin decreased the phosphorylated levels of JNK and p38 in response to Ang II at the indicated time points. ^*^P<0.05, vs. cells at 0 time; ^#^P<0.05, vs. Ang II-treated cells at 0 time. Ang II, angiotensin II; JNK, c-Jun N-terminal kinase.

To further explore the mechanisms underlying the antihypertrophic and antiapoptotic effects of icariin in Ang II-treated H9c2 cells, western blotting was used to detect the phosphorylation levels of JNK and p38, which are key mediators of cardiac hypertrophy and apoptosis. Phosphorylated levels of JNK and p38 were shown to be markedly elevated by Ang II. However, icariin treatment inhibited the phosphorylation of JNK and p38 in response to Ang II at the indicated time points (Fig. 6).

## Discussion

Icariin, the major active component isolated from plants of the Epimedium family, has been reported to exhibit potential protective effects on the cardiovascular system (8,9). However, it is not known whether icariin has a direct effect on Ang II-induced cardiomyocyte injury. In the present study, icariin was found to protect H9c2 cells from hypertrophy and apoptosis in response to Ang II. The beneficial effect of icariin may be mediated by inhibiting the ROS-dependent JNK and p38 pathways.

Enlargement and apoptotic loss of cardiomyocytes play critical roles in the transition from cardiac hypertrophy to heart failure (5). H9c2 cells, an embryonic rat-heart-derived cell line, maintain similar characteristics to primary cardiomyocytes, including morphology, protein expression, electrophysiological properties and hypertrophic responses (15,16). Ang II functions as a significant hormonal mediator in cardiac hypertrophy, which can induce pathological growth and apoptosis in cardiomyocytes (17). In the current study, an Ang II-induced injury model in H9c2 cells was used to evaluate the direct protection of icariin on cardiomyocytes. A previous study demonstrated that icariin attenuated cardiac remodeling in rats with congestive heart failure by inhibiting MMP activity and cardiomyocyte apoptosis (8), indicating the protective role of icariin in the cardiovascular system. However, whether icariin can provide a direct benefit on cardiomyocytes and the underlying mechanisms remain unclear. The results of the present study revealed that icariin directly repressed Ang II-induced H9c2 cell enlargement and the expression of ANP and BNP, which are considered to be molecular markers of cardiomyocyte hypertrophy. Furthermore, icariin blocked apoptosis in Ang II-treated H9c2 cells by regulating the protein expression of the proapoptotic proteins, Bax and cleaved-caspase 3, as well as the antiapoptotic protein, Bcl-2.

Clinical and experimental studies have provided substantial evidence that ROS production, reflecting the status of oxidative stress, is enhanced in hypertrophic and failing hearts (18,19). Ang II, norepinephrine and mechanical stretch can induce the production of ROS associated with the hypertrophic response in cardiomyocytes (20–22). Increased intracellular ROS levels in a hypertrophic heart contribute to the progression of cardiac remodeling and heart failure. H_2_O_2_, a major source of ROS, directly induces cardiomyocyte hypertrophy and apoptosis *in vitro* (23,24). Therefore, antioxidants that block ROS production exhibit therapeutic potential in treating cardiac hypertrophy. Previous studies have shown that icariin attenuated H_2_O_2_-induced neurotoxicity (25) and inhibited ROS production in LPS-treated microglia (11), demonstrating the antioxidative effect of icariin. In the present study, icariin was shown to block the production of ROS in H9c2 cells, which may mediate the cardioprotective effect of icariin.

Increased ROS levels not only lead to the oxidation and damage of macromolecules, membranes and DNA, but also function as a secondary messengers in cellular signaling (26). Activation of JNK and p38, members of the mitogen-activated protein kinase family, has been reported to be induced by ROS (27). Following activation, JNK and p38 phosphorylate a wide array of intracellular targets, including numerous transcription factors, resulting in the reprogramming of cardiac gene expression, the hypertrophic phenotype and apoptosis of cardiomyocytes (28). Inhibition of the JNK or p38 pathways in cardiomyocytes results in attenuated hypertrophic growth induced by agonist stimulation (29). Previous evidence revealed that Ang II-induced activation of JNK and p38 in cardiomyocytes is ROS-dependent (10), indicating that treatment targeting the production of ROS may suppress the JNK and p38 pathways and subsequently result in a protective effect on Ang II-induced injury in cardiomyocytes. The results of the present study demonstrated that icariin downregulated ROS levels and the phosphorylation of JNK and p38 in Ang II-treated H9c2 cells. These observations indicate that icariin protects H9c2 cells from Ang II-induced hypertrophy and apoptosis via the inhibition of the ROS-dependent JNK and p38 pathways.

In conclusion, the current study has demonstrated a previously unknown effect of icariin on Ang II-induced cardiomyocyte hypertrophy and apoptosis through inhibiting the ROS-dependent JNK and p38 pathways. The results of the present study provide experimental evidence for the application of icariin in the treatment of cardiac hypertrophy.
